# Preschool Executive Functioning and Child Behavior: Association with Learning Prerequisites?

**DOI:** 10.3390/children8110964

**Published:** 2021-10-26

**Authors:** Costanza Ruffini, Gian Marco Marzocchi, Chiara Pecini

**Affiliations:** 1 Department of Education, Languages, Intercultures, Literatures and Psychology (FORLIPSI), University of Florence, Via di San Salvi 12, 50135 Firenze, Italy; costanza.ruffini@unifi.it (C.R.); chiara.pecini@unifi.it (C.P.); 2 Department of Psychology, University of Milan-Bicocca, Piazza Ateneo Nuovo, 1, 20126 Milan, Italy

**Keywords:** executive functions, learning prerequisites, working memory, inhibition, self-regulation, behavior, child

## Abstract

Preschool age is a golden period for the emergence of executive functions (EFs) that, in turn, predict learning and adaptive behavior throughout all life. The study was aimed to identify which EFs measures significantly explained the learning prerequisites and the mediation role of self-regulatory and executive behavior recorded in structured or free settings. One hundred and twenty-seven preschoolers were remotely assessed by standardized tests of response inhibition, working memory, control of interference, and cognitive flexibility. Teachers provided a global measure of learning prerequisites by an observational questionnaire. Self-regulatory behavior during the assessment was evaluated by a rating scale filled by the examiners. Executive function behavior in daily life was measured by a questionnaire filled by parents. Accuracy in tasks of response inhibition and working memory explained about 48% of the variability in learning prerequisites while response speed and accuracy in the control of interference and in cognitive flexibility were not significant. EFs also had indirect effects, mediated by the child’s self-regulatory behavior evaluated during the assessment but not in daily life. The results are interpreted with respect to the contribution of the main EF components to school readiness and the mediation of the child behavior as measured in structure contexts.

## 1. Introduction

### 1.1. Executive Functions: Definition and Developmental Trajectories

Executive functions (EFs) refer to cross-modal cognitive processes necessary for goal-directed and adaptive behaviors [1,2,3], able to early predict learning, academic success, health, well-being, economic status, and social actions across life [4,5,6,7].

Although different types of models are used to describe EFs in adulthood and children, fractional models have a great resonance in studying developmental ages. Diamond [3] and Miyake et al. [8,9] describe three main distinct but interrelated basic EFs components. While in Diamond’s model the three components are multi-componential systems, called inhibition, working memory, and cognitive flexibility, Miyake’s model focuses on specific processing operations defined as inhibition, updating, and shifting. Inhibition is the ability to resist temptation, not act impulsively, to override irrelevant stimuli or impulses. In Diamond’s model, inhibition is described as a complex component consisting of the interference suppression, i.e., the ability to focus selective attention on an element surrounded by interfering elements which must be inhibited, and the response inhibition, linked to behavioral self-control, i.e., the ability to resist giving a habitual response in favor of a new and not automatized one. Working memory is a mental space in which verbal or visual-spatial information is manipulated (updated) while it is temporarily held in short-term sores. Cognitive flexibility allows to quickly change (shift) actions and thoughts according to unexpected challenges; it is the ability to think “out of the box” by looking from different points of view [3,8]. All three basic components are assumed to work in different modalities of processing and across different outputs (e.g., visual, verbal, motor, etc.).

EFs develop until late adolescence [10] but the greatest changes are documented during preschool age during which EFs contribute and interact with the development of self-regulation skills, which refers to the children’s ability to regulate their own cognitive, emotional, and behavioral states [11]. The preschool period is characterized by the rapid development of the cognitive and neurofunctional organization of EFs [12]. Many studies support that a progressive differentiation of the executive domain occurs during preschool and is characterized by the emergence of inhibition, working memory, and later, cognitive flexibility [3,13,14,15,16,17]. In turn, the three basic components support the development of more complex and high-level EFs that constitute fluid intelligence, such as abstract reasoning, problem solving, and planning [3,18,19]. EF changes at the cognitive level are associated with the neurofunctional plasticity of development. As cerebral plasticity is highly affected by environmental experiences, it is since the third year of life when the child has acquired the main motor and language milestones and starts to actively move across familiar and extra-familiar contexts and experiences, that the major modifications in the EFs neural network emerge [20]. The preschool period, indeed, is characterized by the strong modulation of the connectivity between the prefrontal cortex, mainly deputed to EFs, and several cortical and subcortical areas deputed to both EFs and other skills [21,22].

Traditionally, the assessment of the executive domain makes use of a series of standardized tests, extensively studied in the literature and with good internal validity [12], which allow to identify the components of EFs and to analyze them individually. Among the tests most commonly and frequently used in preschool age, response inhibition has been measured by the Go-NoGo test [23,24], cognitive flexibility by the Dimensional Change Card Sort test [25,26], suppression of interference by the Flanker test [27,28], and working memory by the Mr. Ant test [29]. Often these tests are administered together or are part of complex batteries [30,31,32] to fully detect the executive domain, respecting the most widespread theoretical models [3,8] and to monitor EF development during childhood.

Given the role of EFs across different modalities of processing and the high cognitive and neurofunctional malleability of EFs characterizing preschool years, it is of paramount importance the correct identification of the preschool EF measures supporting school learning and academic success. 

### 1.2. Executive Functions and Academic Learnings

It is well known that since the first years of primary schools, both literacy and math learning are strongly related to executive functioning [33,34,35,36,37,38,39] as well as to self-regulatory behaviors [40,41,42], so that low skills in these two domains may account for school and learning difficulties [7]. Longitudinal studies found that preschool EFs are general domains predicting later academic acquisition [43,44,45,46,47,48,49]. It has been suggested that during the first years of primary school the child is faced with new and challenging tasks, such as the acquisition of school behavioral rules as well as new literacy and math tasks [50] whereas, as the child progresses through school, behavioral demands tend to reduce so that academic skills may rely more on automatized, domain-specific knowledge and less on executive control [51,52,53]. 

The relationship between EFs and school readiness could start before primary school, in the acquisition of learning prerequisites. Learning prerequisites refer to a complex domain involving cognitive, motor, and language milestones as well as behavioral and emotional aspects [54]. They may be measured in terms of specific school competencies (e.g., rapid visual naming, number recognition, phonological awareness, letter knowledge, etc.) or as a global index merging the mentioned specific skills with more general school attitudes (e.g., paying attention, respecting rules, etc.). Although transversal skills, such as EFs, linked to different learning domains, can represent relevant prerequisites for school readiness and academic success [55], cultural differences in educational policy across countries and environmental conditions (e.g., urban area, suburbs, or rural [56]) could affect the prerequisites directed by the educational programs and the strategies used to promote them [57,58,59].

The literature suggests a significant relationship between preschool EFs and learning prerequisites [60,61,62,63,64,65,66,67], nevertheless, the individual contribution of the basic EF components and the interactive role of self-regulatory behaviors on global learning prerequisites, is not completely defined.

EFs may support learning prerequisites and school readiness at both cognitive and behavioral levels. At the cognitive level, the abilities to control impulsive behavior and interference stimuli, to upload information held in short-term memory and to change strategies or responses, represent mental operations needed in the first phases of alphabetization, when literacy and math are not automatized at all, nor they may rely on intuitive acquisition strategies [45,53]. Furthermore, as Gunzenhauser and Nuckles, [68] have suggested, EFs can support academic skills by a “learning-related behaviors’’ way, that is by supporting behaviors that are appropriate to the school context, such as maintaining attention in the classroom, resisting distractions, finishing activities, following the rules of the task. The “learning-related behaviors” are indeed self-regulatory behaviors within the school context. Self-regulation consists of a multiplicity of processes that allow the individual to control his own thinking (cognitive self-regulation), his own behavior and acting (behavioral self-regulation), and his own emotional reactions within social interactions (emotional self-regulation) in order to achieve a set goal [69]. Although cognitive development is hardly discernible in separate and modularized components, especially in early ages [70], EFs are suggested to support self-regulation since preschool [71,72]. Historically, self-regulation in the developmental age is mainly detected through assessments by an adult who observes the child in their natural context, such as home or school while EFs are assessed directly from the behavior emitted by the child through standardized experimental tests far from the real world [3,11]. The involvement of EFs in self-regulation processes could support a mediator role of self-regulation between EFs and learning prerequisites.

Thus, defining which behaviors can mediate the relationship between EFs and learning prerequisites is crucial for intervention but, in fact, it is a matter of debate and needs studies from different cultures and educational systems. Recently, there have been developed several questionnaires and rating scales to ecologically measure self-regulatory and executive control behaviors in daily life across different ages [73,74]. These types of instruments are extremely relevant for preschoolers as they may be rather unfamiliar with the structured evaluations and demands required by standardized cognitive tests. Nevertheless, the performances at structured EF tests and the self-regulation and executive functioning behaviors, measured at rating scales, do not correlate, and seem to capture different processes [75,76]. As Toplak and collaborators [77] suggest, performance measures at cognitive tests may reflect the individual’s processing efficiency in structured settings whereas rating scales reflect an individual’s ability to accomplish goals in unstructured settings. According to this interpretation, the absence of correlation mentioned above may be in part due to the different contexts investigated, structured context, and daily life respectively, as the former can be characterized by stricter and more structured demands. 

Thus, while it is expected that the relationship between EFs and learning prerequisites is mediated by self-regulatory behaviors held by the child during structured tasks within institutional settings, reflecting more the “school learning-related behavior”, self-regulatory and executive functioning behavior observed by parents in unstructured daily life may not mediate the relationship between EF skills and learning prerequisites.

### 1.3. The Present Study

The present research examines which different components of Executive Function are related to learning prerequisites in children attending kindergarten and if there is a mediation role of the behavior in structured and unstructured settings. In particular: (1) which are the basic EFs, among response inhibition, interference control, cognitive flexibility, and working memory, that significantly explain the variability in learning prerequisites measured by teachers? (2) Is the behavioral observation during the execution of a task a significant mediator between the EF task performance and learning prerequisites? (3) Is the executive behavior observed by parents in the daily activities a significant mediator between the EF task performance and learning prerequisites?

Our hypothesis is that all EF basic components could explain individual variability in learning prerequisites defined in terms of a global index merging several skills, such as motor coordination, language comprehension and expression, reasoning, metacognition, and pre-alphabetization. Secondly, we predict that the observed behavior on the executive tasks could be a significant mediator between EFs and learning prerequisites, whereas the daily executive function behavior rated by the parents could be not a significant mediator between the EF task performance and learning prerequisites.

## 2. Material and Method

The project was approved by the Ethical Committee of the University of Florence (protocol n. 87, 22 May 2020) and was carried out following the Ethical guidelines of the Italian Association of Psychology. Five preschools in Tuscany (Italy) consented to participate in the project in December 2020. 

### 2.1. Participants

One hundred and twenty-seven children took part in the study; age ranged between 39 and 74 months (Mean = 61.17; SD = 9.24; 71 Males). For all children, parents’ written consent and children’s verbal consent were obtained. Socioeconomic and cultural information were recorded by a questionnaire filled by parents. All children were native to the Italian language (L1) and 34 children (26.7%) were systematically exposed to a second language. No child had a diagnosis of Neurodevelopmental Disorder. The socio-economic status (SES) was calculated (for all but one orphaned child) by adding the employment of the mother and the father (working or unemployed) and the level of education of both parents (elementary school, middle school, diploma, degree). SES (N = 126; Mean = 8; SD = 1.47) ranged from a minimum of 2 (unemployed and elementary school parents) to a maximum of 10 (working and graduated parents). 

### 2.2. Assessment Procedure

EFs were measured by a remote assessment lasting approximately 20–30 min. During the weeks of assessment, teachers completed a questionnaire on the child’s learning prerequisites, the experimenters filled a rating scale on the child’s behavior during the assessment and parents completed a questionnaire related to self-regulatory and executive function behavior in daily life.

#### 2.2.1. Remote Assessment of EFs

The remote assessment was conducted by the Gorilla Experiment Builder™ (https://app.gorilla.sc/ from 25 January 2021 to 16 April 2021), which is a platform that allows the creation and the remote use of behavioral experiments. The Gorilla.sc platform requires an internet connection and is accessible from any browser (in particular Safari, Chrome, and Samsung Browser were used) and from any device (in particular Apple iPad, Samsung SM-T515, and Samsung SM-T8T19, ranging between 9.7 and 12 inches, were used).

Apart from one preschool, where there was a Wi-Fi connection, the Hot Spot connection from a mobile device was used.

An in-presence operator adequately prepared the room within the preschool building where the remote assessment was conducted, placed a tablet on an empty table, and a computer behind the child and made sure that the room was quiet enough and with adequate brightness for using digital devices. The in-presence operators were adequately prepared and received instructions on the correct procedure for the remote assessment (e.g., child and tablet’s positioning, behavioral rules solutions for technological problems, etc.). During the assessment, the child sat about 30–50 cm from the tablet and the in-presence operator sat next to the child to ensure physical safety, to favor a correct position of the child in front of the tablet, and to respond to any child’s exceptional needs (e.g., drinking, going to the bathroom, etc.).

Connected to the in-room computer, a remote psychologist in training, observed the entire session of the assessment and rated the child’s behavior. The remote psychologist kept the camera and microphone off so as not to disturb the child during the assessment.

The remote assessment of EFs consisted of four tests, whose audio, video instructions, and answers were automatically presented and recorded by the software. The following tests were proposed according to a fixed order.

- Go–NoGo test (GnG, modified from Donders [78])

This test measures response inhibition [23,79]. The child sits in front of the tablet and watches two images (Go: picture of a banana, 1150 × 1147 pixels; NoGo: picture of a watermelon, 660 × 847 pixels), he/she is instructed to respond by touching the screen for the Go stimuli and to not respond for the NoGo stimuli. The test consists of 3 blocks of 25 items each; in each block, the proportion of Go stimuli is 30%. Each stimulus is presented until the child responds for a maximum of 1000 ms with an interstimulus interval of 500 ms. The number of correct responses (CR) to Go stimuli (from 0 to 57) and to NoGo stimuli (from 0 to 18) and median reaction times (T) for the Go stimuli are recorded.

- Dimensional Change Card Sort test (DCCS, modified from Zelazo [25])

The Dimensional Change Card Sort Test measures shifting processes [31]. In order to perform the task, the child must be able to classify the cards according to color (red or blue) or shape (rabbit or boat) criteria.

**Figure 1:**
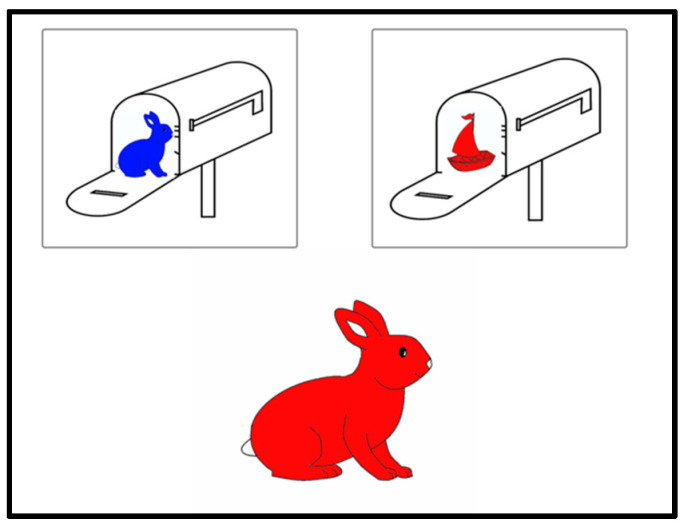
Example of the stimuli used for the Dimensional Change Card Sort test. Top: pictures of the two letter-boxes; bottom: an example of the stimulus target.

Twenty-four pictures (about 961 × 1159 pixels) representing red or blue rabbits and red or blue boats and two depicted letter-boxes representing a blue rabbit and a red boat are used (Figure 1). The task is composed of three conditions: (1) Color condition (6 pictures): the child has to indicate the letter-box representing the red boat when a red card (boat or rabbit) appears on the screen and the box representing the blue rabbit when a blue card (boat or rabbit) is shown; (2) Shape condition (6 pictures): the child has to indicate the box depicting the blue rabbit when a card with a rabbit (red or blue) is presented and indicate the box depicting the red boat when a boat (red or blue) is presented; (3) Border condition (12 pictures): the child is instructed to follow the rules of the Color condition when the picture has a black border and the rules of the Shape condition when the picture has not a black border. The number of correct responses to the Shape condition was used as a measure of cognitive flexibility since it requires the shift from the first to the second classification criteria.

- Flanker test (F, modified from Eriksen and Eriksen [80])

**Figure 2:**
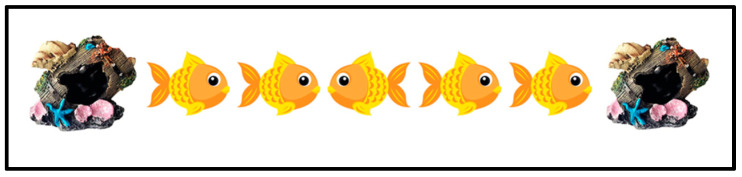
Example of the stimuli used in the Flanker test (incongruent condition).

This task measures the interference control: the capacity to inhibit irrelevant information. The picture (939 × 751 pixels) of five fish lined up is presented on the screen. In 26 trials, all the fish look towards the same (left or right) direction (congruent condition), in 26 trials, the fish in the center looks the opposite direction in comparison to the other lateral 4 fish (incongruent condition). The child has to decide as soon as possible the direction of the fish in the center of the screen (target fish) and to touch the cave on the right if the target fish is looking to the right or on the left if it is looking to the left (Figure 2). The test consists of 4 practice items (2 congruent and 2 incongruent) and two blocks of 24 items each. In each block there are 12 congruent items and 12 incongruent items; each item is presented for 5000 ms with a random inter-stimulus interval ranging between 400 ms and 1200 ms. The maximum response time is set to 5000 ms. The number of correct responses (CR, from 0 to 26) and median reaction times (T) are recorded both for the congruent and incongruent conditions.

- Mr. Giraffe test (MG, modified from Morra [29])

**Figure 3:**
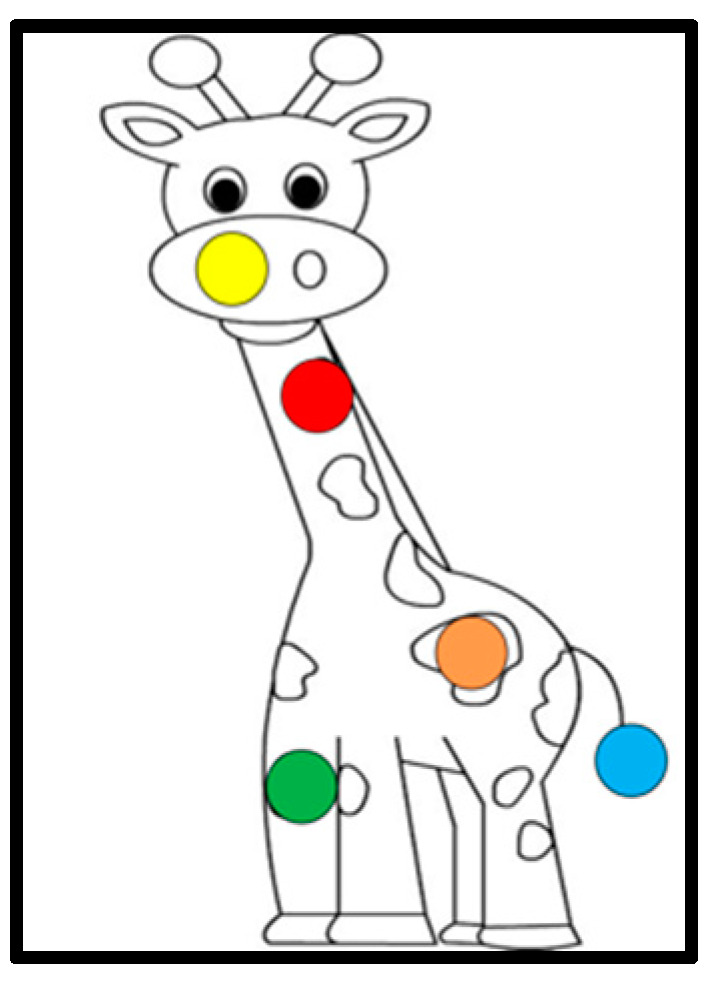
Example of the items used in the Mr. Giraffe test (5 spots—condition).

This test evaluates visuospatial working memory [31]. The picture of a giraffe (about 960 × 1179 pixels) is presented on the tablet screen; an increasing number of spots (89 × 91 pixels) appear on the giraffe (Figure 3). The number of spots ranges, on 3 consecutive trials, from 2 to 6, for a total of 15 trials. Each picture is presented for 5 s (items 1–12) and for 6 s (items 13–15). Immediately after the picture presentation, the child has to indicate or touch the position of all spots on a blanked picture of the giraffe. The number of correct responses is recorded.

#### 2.2.2. Assessment of Learning Prerequisites

- IPDA questionnaire [81]

The IPDA is a standardized questionnaire, filled by teachers after observing the child for at least one week, addressed to preschoolers’ prerequisites. It is composed of 43 items related to general learning predisposition skills, that consist of behavioral school attitude (e.g., following instructions and rules), fine motor skills (e.g., general movement coordination), language comprehension (e.g., understanding the meaning of the words used by teachers), oral expression (e.g., using a rich vocabulary), metacognition (e.g., focusing on objectives and strategies), memory (e.g., memorizing short nursery rhymes), praxis (e.g., drawing a human figure with the main body parts), orientation (e.g., orienting well and readily in space) and specific skills, related to the prerequisites of literacy (e.g., understanding that words are composed of separate sounds—phonemes) and math (e.g., connecting quantities to digits). Summing all the items’ scores, this instrument provides a global, ecologic but not fine score indicating the level of a child’s learning prerequisites. The questionnaire was standardized on an Italian sample and demonstrated a good reliability and validity. For each child, teachers are required to rank each item on a 5-point Likert scale (from “not at all” to “a lot”). The scale takes about 15 min. Final scores range from 43 (lowest prerequisites) to 215 (highest prerequisites).

#### 2.2.3. Assessment of the Self-Regulation and Executive Functioning Behavior

- Self-Regulatory behavior during Structured Activities (SR-SA, modified from Sutherland [82])

This rating scale provides information on the behaviors adopted by the child during activities in a structured assessment situation. The remote observer (a trainee psychologist) was connected to the room using Google Meet, observed the child’s behavior and her/his comments or requests, and filled the scale immediately after the child assessment was ended. The computer, with the camera and microphone off, was placed on a table behind the child in an appropriate position so that the remote operator could correctly observe the child. The items refer to the following aspects: compliance, activity level, restlessness, necessity of breaks, attention, distractibility, type of distractors, adequate behavior, frustration, fatigue, anxiety, requests of confirmation, and support. Each item is scored on a Likert scale ranging from 1 to 3 (items 1–4, 6–8, 12) or from 1 to 4 (items 5, 9, 10–11); total scores range from 12 (regulated behavior) to 40 (dysregulated behavior). For the Italian adaptation, inter-judge agreement, measured on 47 children of the sample, was 85%.

- Executive Function Behavior (EFB [83])

This rating scale provides information about children’s control processes and Executive Function Behavior in daily life according to the parents. It includes 24 items, on a Likert scale ranging from 1 to 5. Items belong to different aspects such as cognitive self-regulation (e.g., when given two things to do, he/she remembers them both), behavioral self-regulation (e.g., he/she is able to behave in a controlled way), material management (e.g., when he/she is instructed to tidy up and has figured out what to do, he/she does everything carefully and orderly), flexibility/adaptation (e.g., he/she easily adapts to pre-established routines for play, sleep, nutrition) and initiative (e.g., when he/she wants to start a task, he/she does it without having to repeat it). A total score ranging from 24 (poor self-regulation) to 120 (high self-regulation) is obtained.

### 2.3. Statistical Analysis

Statistical analyses were conducted by the Statistical Package for Social Science 2021, version 27.0 (SPSS, IBM Corporation), and by Jamovi, version 1.6.23.

Descriptive statistics, analysis of normality of the score distributions, and parametric bivariate correlations were conducted on all variables. As data were nested in classes, ICCs values were calculated for each variable. On the IPDA subscales scores, a principal component analysis (Varimax method) was conducted in order to guarantee the appropriateness of using a global score of learning prerequisites.

Multiple linear regression and the multicollinearity analyses were run in order to identify the EF measures (number of correct responses and time to the Go items and number of correct responses to the NoGo items at the Go–NoGo test, number of correct response to the Dimensional Card Sorting test—Shape condition, number of correct responses and time to the congruent and to the incongruent items at the Flanker test, number of correct responses to the Mr. Giraffe test) that explained learning prerequisites at the IPDA questionnaire. Age and socio-economic status were introduced in the model as covariates.

To explore how each significant EF predictor of learning prerequisites was mediated by children’s behavior, mediation models with SR-SA and EFB scores as independent mediators were tested by the PROCESS macro in SPSS.

Since the tools used are drawn from standardized tests that detect specific EFs and are widely used in preschool, the tests’ measures were used directly for the regression and mediation analyses.

## 3. Results

Descriptive statistics of age, socio-economic status, performances on the EF tests, and rating scales are reported in Table 1.

A percentage of children were unable to complete the Go–NoGo (4.7%) and the Flanker (9.4%) tests due to the difficulties to understand and follow the instructions.

Visual inspection of the data shows a high variability of the performances, especially in the response time at the Go–NoGo and Flanker tests. The principal component analysis supported a unique factor underlying the IPDA subscales (fitting values ranged between 0.85 and 0.97).

Analysis of the normality of the distributions, reported in Table 2, show that all variables are normally distributed according to a cutoff of 2 for skewness and 3 for kurtosis [84,85].

Results of the bivariate correlational analysis among all variables (see Table 3) show that almost all measures of EFs are positively correlated among each other, the IPDA is significantly correlated with all measures of accuracy at the EF tests and the SR-SA and EFB scores, the SR-SA rating scale is significantly correlated with 6 out of 8 EFs measures while the EFB is significantly correlated to one EF measures and to the IPDA score. Age significantly correlated with all variables except for Go–NoGo median time and socio-economic status. Socio-economic status significantly correlated with Go–NoGo median time, learning prerequisites, and behavioral questionnaire.

The linear regression analysis of the EF variables on the learning prerequisites at the IPDA revealed that the number of correct responses to the Go (β = 0.29, *p* < 0.01) and NoGo stimuli (β = 0.32, *p* < 0.001), and the number of correct responses at Mr. Giraffe test (β = 0.3, *p* < 0.01) were significant predictors. Reaction time for the Go–NoGo test, the number of correct responses, and the reaction time for congruous and incongruous conditions for the Flanker test and the correct responses for the Dimensional Change Card Sort Shape condition were not significant predictors. Socio-economic status (β = 0.19, *p* < 0.05), as covariate, significantly predicted learning prerequisites. Multicollinearity was rejected for all variables. The final regression model significantly explained 48% of the variance of the IPDA score (R^2^ = 0.48, F(11, 97) = 8.07, *p* < 0.001). Regression coefficients for all predictors and covariates are shown in Table 4.

**Figure 4:**
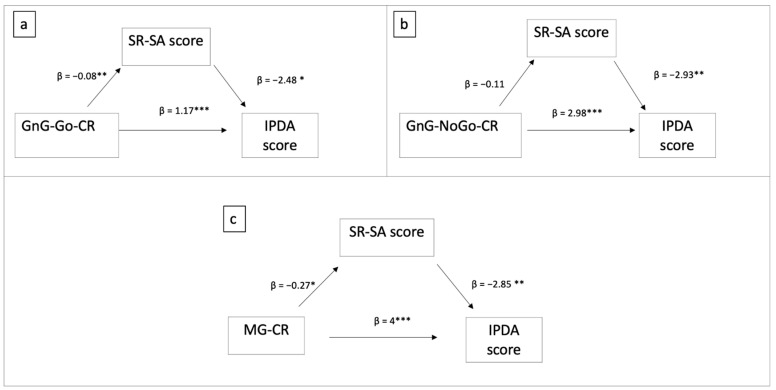
Direct and indirect effect of the EFs ((**a**): correct responses at Go-NoGo Test Go condition; (**b**): correct responses at Go-NoGo Test NoGo condition; (**c**): correct responses at Mr. Giraffe test) on the IPDA score via the Self-regulation behavior during structured activities scores. * *p* < 0.05; ** *p* < 0.01; *** *p* < 0.001.

In agreement with the study aims, for each significant EF predictor of the IPDA scores, a mediation model was run with the scores at the SR-SA and EFQ scales as independent mediators. Figure 4 describes the models obtained when the SR-SA scores were used as mediators.

As shown in Figure 4a, the number of correct responses to the Go condition significantly explained the variability in IPDA scores both directly (b = 1.17, s.e. = 0.29, *p* < 0.001) and indirectly via the SR-SA scale scores (path 1: b = −0.08, s.e. = 0.03, *p* < 0.01; path 2: b = −2.48, s.e. = 1.02, *p* < 0.05; indirect path: b = 0.19, bootstrap 95%, s.e. = 0.11, C.I. [0.02:0.45]). Overall, the focal predictor plus the mediator explained 19.95% of the variability observed in the IPDA (F(2, 118) = 14.71, *p* < 0.001). The total effect of GnG–Go–CR on the IPDA was significant (b = 1.36, s.e. = 0.29, *p* < 0.001).

The number of correct responses to the NoGo condition (Figure 4b) significantly explained the variability in IPDA scores both directly (b = 2.98, s.e. = 0.7, *p* < 0.001) and indirectly although the effect of GnG–NoGo–CR on SR-SA was not significant (path 1: b = −0.11, s.e. = 0.06, *p* > 0.05; path 2: b = −2.93, s.e. = 0.99, *p* < 0.01; indirect path: b = 0.32, bootstrap, s.e. = 0.2, 95% C.I. [0.01:0.79]). Overall, the predictor plus the mediator explained 21% of the variability observed in the IPDA (F(2, 118) = 15.68, *p* < 0.001). The total effect of GnG–NoGo–CR on the IPDA was significant (b = 3.3, s.e. = 0.72, *p* < 0.001).

The number of correct responses at the Mr. Giraffe test (Figure 4c) significantly explained the variability in IPDA scores both directly (b = 4, s.e. = 1.1, *p* < 0.001) and indirectly via SR-SA scale (path 1: b = −0.27, s.e. = 0.11, *p* < 0.05; path 2: b = −2.85, s.e. = 0.89, *p* < 0.01; indirect path: b = 0.77, bootstrap 95%, s.e. = 0.37, C.I. [0.13:1.6]). Overall, the predictor plus the mediator explained 19.40% of the variability observed in the IPDA (F(2, 124) = 14.93, *p* < 0.001). The total effect of the scores at the Mr. Giraffe test on the IPDA was significant (b = 4.77, s.e. = 1.12, *p* < 0.001).

**Figure 5:**
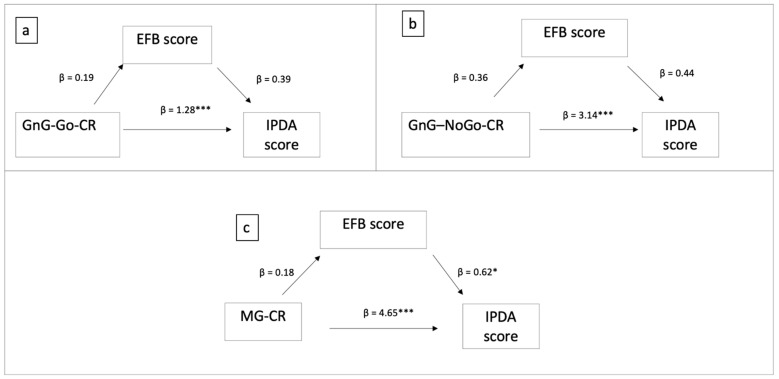
Direct and indirect effect of the EFs ((**a**): correct responses at Go-NoGo Test Go condition; (**b**): correct responses at Go-NoGo Test NoGo condition; (**c**): correct responses at Mr. Giraffe test) on the IPDA score via the Executive function behavior scores. * *p* < 0.05; *** *p* < 0.001.

Figure 5 describes the models obtained when the EFQ scores were used as mediators.

As shown in Figure 5a, the number of correct responses to the Go condition significantly explained the variability in IPDA scores directly (b = 1.28, s.e. = 0.29, *p* < 0.001) but not indirectly (path 1: b = 0.19, s.e. = 0.11, *p* > 0.05; path 2: b = 0.39, s.e. = 0.24, *p* > 0.05; indirect path: b = 0.08, bootstrap 95%, s.e. = 0.07, C.I. [−0.04:0.23]). Overall, the focal predictor and the mediator explained 17.81% of the variability observed in the IPDA scores (F(2, 118) = 12.78, *p* < 0.001). The total effect of GnG–Go–CR on the IPDA scores was significant (b = 1.36, s.e. = 0.29, *p* < 0.001).

The number of correct responses to the NoGo condition (Figure 5b) significantly explained the variability in IPDA scores directly (b = 3.14, s.e. = 0.72, *p* < 0.001) but not indirectly (path 1: b = 0.36, s.e. = 0.27, *p* > 0.05; b = 0.44, s.e. = 0.24, *p* > 0.05; indirect path: b = 0.16, bootstrap 95%, s.e. = 0.16, C.I. [−0.07:0.54]). Overall, the focal predictor and the mediator explained 17.44% of the variability observed in the IPDA scores (F(2, 118) = 12.46, *p* < 0.001). The total effect of the correct answers to the NoGo on the IPDA scores was significant (b = 3.3, s.e. = 0.72, *p* < 0.001).

The number of correct responses at the Mr. Giraffe test (Figure 5c) explained the variability in the IPDA scores directly (b = 4.65, s.e. = 1.09, *p* < 0.001) but not indirectly (path 1: b = 0.18, s.e. = 0.39, *p* > 0.05; path 2: b = 0.62, s.e. = 0.25, *p* < 0.05; indirect path: b = 0.11, bootstrap 95%, s.e. = 0.24, C.I. [−0.33: 0.65]). Overall, the focal predictor and the mediator explained 16.97% of the variability observed in the IPDA scores (F(2, 124) = 12.67, *p* < 0.001). The total effect of working memory on the IPDA scores was significant (b = 4.77, s.e. = 1.12, *p* < 0.001).

## 4. Discussion

The present study investigated the relationship between basic EF components and learning prerequisites in typically developmental preschoolers to define which EF measures have a significant role in explaining school readiness and whether such a role is mediated by the child’s self-regulating behavior during the execution of the tasks or in the daily home activities.

For that purpose, a sample of preschoolers aged between 3 and 6 years was evaluated for EF skills, learning prerequisites, self-regulatory and executive behavior. EF tests were selected on the basis of the literature that demonstrated they were valid measures of the EF components emerging during preschool [31,32]. Learning prerequisites were evaluated by a standardized rating scale filled by teachers providing an omni comprehensive measure of the cognitive processes and behavioral readiness required for school learning. Self-regulatory and executive behavior was contemporarily measured in two different contexts, on the basis of the hypothesis that the role of EFs on school learning is mediated by the behavior held by the child in structured settings, such as that one of the assessments, rather than in free and unstructured settings such as the daily life.

The first goal of the current study was to test which EF components and measures would significantly explain the learning prerequisites measured by a rating scale filled by teachers. Among all measures of EFs, the accuracy of response inhibition and visuo-spatial working memory significantly explained almost 48% of the inter-individual variance in learning prerequisites. This result generally confirms the vast literature supporting the role of EFs on learning prerequisites [60,61,62,63,64,65,66,67] and suggests that educational strategies and interventions targeted to empower the basic EF components in preschoolers could strongly affect general school prerequisites and readiness [66]. In Brock and colleagues’ study [61], EF cool components (those processes that operate in neutral, non-emotional contexts usually comprehending the basic EFs components), both as a composite score and as single measures (i.e., motor coordination and inhibition), predicted early math, but not literacy. In Clark and colleagues’ study [63], EF composite score (unifying measures of working memory and of inhibitory control) at age 3 years predicted 41% of the variance of general math proficiency at age 5.3. Bull and colleagues’ study [62] confirmed the predictive association between executive control and math. Miller and colleagues [64] found that working memory, but not inhibition, explained 52% of the variance in literacy and 81% in math. Traverso and colleagues [66] identified a significant effect of the interference suppression score on the math performances and on the improvement in reading and writing skills after an EF training. Howard and colleagues [67] demonstrated that an EF composite score (summing working memory, inhibition, and flexibility), together with scores of cognitive and behavioral self-regulation, predicted consistently advanced learning, measured in terms of school readiness. In Willoughby and colleagues’ study [65], preschoolers with poor EFs, in contrast to children with high EFs (in terms of working memory, inhibition, flexibility), showed impairments in multiple indicators of academic readiness. Nevertheless, the mentioned studies differ for the EF components found to be predictive and, in comparison to our results, some of them did not find the predictive role of working memory and response inhibition [64,66]. To interpret such a difference, the type of learning prerequisites measured and the developmental trajectories of EFs must be considered. While previous studies focused on literacy and math prerequisites, the present study measured learning prerequisites as a general predisposition attitude, encompassing several cognitive and behavioral processes, such as fine motor skills, language, metacognition, memory, orientation, pre-literacy, and pre-math [81]. This type of measure may be more related to those EF components, such as response inhibition and working memory, that develop early and have already emerged in preschool age while other EFs such as interference control and, above all, cognitive flexibility tend to emerge later [14,15,16,17], may be more related to the acquisition of specific and fine skills that are subsequently acquired during primary school, such as literacy and math. Indeed, in our study, the performances at the control of interference and the cognitive flexibility tests did not explain variability in learning prerequisites. Moreover, the interference control task showed the highest percentage of missing data because children found it too difficult, as well as measures of speed, reflecting automatization of control processes, showed a high inter and intra-subject variability and none were significant. Further studies gathering the sample age in different ranges and separating the measures of general and specific learning prerequisites could help in verifying such an interpretation. Moreover, it is also important to underline the significant role of the socioeconomic level, introduced in the model as a covariate, in explaining the learning prerequisites; coming from a higher socio-economic level seems to be protective factors for the development of school learning. This is in line with ample evidence in the literature that low SES represents a risk factor for cognitive development and learning [86,87,88].

The second goal of the study was to test the hypothesis that the self-regulatory behavior observed during the EF tasks execution could mediate the relation between the EF measures and the learning prerequisites. The hypothesis was confirmed by the mediation analysis showing that response inhibition and working memory favored school readiness both directly and indirectly through self-regulatory behavior: the highest response accuracy in Go and NoGo trials and the number of items remembered in the working memory task, the highest were the self-regulation scores obtained during the assessment and, in turn, the levels of scholastic preparation evaluated by teachers.

The third goal of our study was to analyze whether the executive behavior evaluated by parents in a daily life context could be a significant mediator of the relationship between EF performance and learning prerequisites. As hypothesized, the daily executive behavior at home, as rated by parents, showed weak correlations with the measures at the EF tests and it was not a significant mediator of the relationship between EFs and learning prerequisites.

Altogether, these results confirm and extend the previous literature providing useful methodological and educational insights.

For what concerns the methodological aspects, the scientific literature and clinical experience in the domain of EFs suggest that there is often a low correlation between the results of standardized tests and those of questionnaires [75,76,77]. The tests generally evaluate the efficiency of a process in an optimal and structured situation: the assessed child is asked to carry out a task individually, following defined instructions, when goals are clearly defined from the outside, in an environment generally designed for this purpose and therefore quiet and facilitating. The scales and questionnaires evaluate behaviors in everyday life and the parent, for example, must evaluate how the child usually behaves in a usual and unstructured situation when goals are instead defined by the individual himself without explicit guidance. According to this view, it is therefore not surprising the inconsistency between direct and indirect evaluation tests of EFs that should not be considered as equivalent and interchangeable measures [77]. Our study extends this perspective by suggesting that it is not the tool itself, test vs observational questionnaire, but rather the context in which the child is observed that matters. The results of the mediation analysis conducted in our study show, indeed, that the “learning-related behaviors’’ way by which EFs support academic skills [68] can be measured by the observation of the self-regulatory behavior held by the child in a structured context, such as the assessment by EF tests. Observing the child’s behavior, when carrying out particular activities during the administration of tests, can provide useful information on the child’s learning behavior at school and on the effect of EFs on it. For example, poor metacognitive control, difficulty in keeping in mind the objectives or rules of a task, distractibility, persistence in the same mistakes and resistance to change strategy or impulsiveness are all possible indicators of difficulty in basic EFs that could correlate, more than what is observed by parents in daily life, with the learning behavior needed in the structured school context. The relationship between EFs and learning behavior in preschool children was also found by Brock and colleagues [61]; however, these authors did not find the mediating role of learning-behavior between EFs and learning prerequisites. Compared to the current study, Brock and colleagues considered, as dependent variables, the performances on specific domains of math and literacy, and not global indexes of learning prerequisites, as in fact it was collected via the IPDA questionnaire in the present study. We suggest that measures of learning prerequisites synthesizing different cognitive and behavioral skills could be more sensible, than specific skills, to general domain factors such as executive functioning and self-regulatory behavior. Moreover, it is worth noting that while in Brock’s study the scales were ranked by teachers, in the present study the behavior rating scale was filled by expert psychologists trained to observe signals of behavioral and cognitive difficulties of the child.

For the educational and interventional implications, the results of our study suggest that preschool programs taking into consideration the interplay among EFs, self-regulatory behavior and learning prerequisites will be promising. In order to favor readiness to learn and chances to be in more academic stimulating environments, preschool children may need both cognitive training on the basic EFs, in particular on inhibition and working memory and opportunities to experiment themselves in self-regulatory behaviors. For the latter, however, it must be considered the context-specific effect found in our study according to which self-regulating behavior could hardly generalize across different contexts. In order to promote learning-behavior attitudes, self-regulatory challenges may need to be proposed in structured school contexts rather than in free contexts that are in fact far from the school requests, without rigid duties to be respected.

## 5. Limits and Strengths of the Study

It is worth mentioning that the present study has some limitations that can be ascribed to the sample and the procedure used. The small sample number, together with the wide age range across all preschool years, prevented any comparison between ages whereas, as mentioned, it could help to describe the developmental trajectories of the EF-learning prerequisites relationship. Furthermore, although our sample included children without neurodevelopmental disorders or special needs, fluid intelligence was not measured while it could represent a further mediator of the EF-learning relationship. Finally, since EFs are considered general domain processes, the modality used for stimuli presentation can be further investigated, for example distinguishing between visuo-spatial and verbal components of working memory.

Another aspect to consider is that the present study used cross-sectional data collected at the same time—it would be interesting to confirm these results with a longitudinal study.

A strength of this study is the use of innovative technology for measuring EFs, a procedure that has proved feasible and well accepted by children. This type of survey is highly reproducible in different socio-cultural and historical contexts, such as the one linked to the COVID-19 restrictions.

## 6. Conclusions and Future Directions

In conclusion, the current study provides evidence that response inhibition and visuo-spatial working memory, but not control of interference and cognitive flexibility can significantly explain inter-individual variability on a composite index of learning prerequisites. Moreover, the behavior observed during the execution of the EF tasks, but not the daily executive behavior observed at home by parents, is a significant mediator of the relation between EFs and learning prerequisites. These results suggest basing the evaluation of EFs not only on a single type of tools or of raters. Firstly, a complete evaluation of EFs in preschoolers needs to integrate direct methods, i.e., structured cognitive tests, focused on EF cognitive processes, with indirect methods, i.e., questionnaires, focused on self-regulatory and executive behavior. Secondly, questionnaires must gather different perspectives and be ranked by the several adult figures taking care of the child, such as examiners, teachers, and parents.

Furthermore, the results of the study have implications for future research and intervention. As the present study was conducted in Italy, it could be important to describe the relationship between EFs and learning prerequisites, through self-regulatory behavior, across different cultural backgrounds and educational policies. Good levels of EFs and successful interventions to empower EFs and self-regulation in preschoolers may be particularly important upon entering the school context to give everyone the opportunity to acquire adequate school skills. It can be suggested that educational actions aimed to promote EFs and learning prerequisites in preschool need to work also through the facilitation of self-regulatory behaviors and that, to such an aim, proposing structured activities in institutional contexts may train children to adopt the learning-behavior approach needed for schools.

## Figures and Tables

**Table 1:** Descriptive statistics of age, socio-economic status the scores at the EF tests, the learning prerequisites, the behavioral questionnaires and scales.

Measure (n)	Mean (SD)	Range (Min–Max)	ICCs
Age in months (127)	61.17 (9.24)	39–74	
SES (126)	8 (1.47)	2–10	
GnG–Go–CR (121)	45.36 (11.17)	6–57	0.06
GnG–T (121)	683.05 (128.23)	396–978	0.02
GnG–NoGo–CR (121)	15.37 (4.48)	0–18	0.05
DCCS–CR (127)	4.12 (1.74)	0–6	0.18
FC–CR (122)	18.46 (6.89)	1–26	0.11
FC–T (121)	1420.14 (444.51)	592.2–2720.3	0.05
FI–CR (121)	12.80 (7.37)	0–26	0.04
FI–T (115)	1564.69 (736.58)	104–3357	0.13
MG–CR(127)	2.20 (2.99)	0–12	0.1
IPDA score (127)	160.84 (39.93)	64–215	0.16
IPDA behavior subscale	33.57 (8.57)	12–45	
IPDA motor skills subscale	7.89 (1.94)	2–10	
IPDA language subscale	12.12 (2.7)	6–15	
IPDA oral abilities subscale	18.67 (5.24)	5–25	
IPDA metacognition subscale	13.79 (4.1)	4–20	
IPDA cognition subscale	38.73 (10.13)	11–50	
IPDA pre-literacy subscale	24.61 (7.99)	0–35	
IPDA pre-math subscale	11.47 (3.38)	3–15	
SR-SA score (127)	14.96 (3.71)	12–30	0.13
EFB score (127)	91.68 (13.18)	60–120	0.00

Legend: ICC: Intraclass Correlation Coefficients; SES: Socio-economic Status; CR = correct responses; T = median time; GnG = Go–NoGo test; Go = Go condition; NoGo = NoGo condition; DCCS = Dimensional Change Card Sort test Shape condition; FC = Flanker test congruous condition; FI = Flanker test incongruous condition; MG = Mr. Giraffe test; SR-SA = Self-regulation behavior during Structured Activities; EFB = Executive Function Behavior.

**Table 2:** Skew and kurtosis of the scores distributions for age, socio-economic status, the EF tests, the learning prerequisites, the behavioral questionnaires and scales.

Measure	Skew	Kurtosis
Age in months	−0.66	−0.36
SES	−0.75	1.06
GnG–Go–CR	−1.74	2.72
GnG–T	0.13	−0.82
GnG–NoGo–CR	−2.05	3.3
DCCS–CR	−0.21	−1.49
FC–CR	−0.48	−1.18
FC–T	0.26	0.08
FI–CR	0.22	−0.94
FI–T	0.53	−0.5
MG–CR	1.13	0.2
IPDA score	−0.48	−0.83
SR-SA score	1.61	2.53
EFB score	−0.00	−0.42

Legend: SES: Socio-economic status; CR = correct responses; T = median time; GnG = Go–NoGo test; Go = Go condition; NoGo = NoGo condition; DCCS = Dimensional Change Card Sort test Shape condition; FC = Flanker test congruous condition; FI = Flanker test incongruous condition; MG = Mr. Giraffe test; SR-SA = Self-regulation behavior during structured activities; EFB = executive function behavior.

**Table 3:** Correlations between all variables.

	1	2	3	4	5	6	7	8	9	10	11	12	13	14
1. Age in months	-	0.02	0.43 ***	0.14	0.36 ***	0.30 ***	0.57 ***	0.24 **	0.24 **	0.33 ***	0.4 ***	0.51 ***	−0.39 ***	0.24 **
2. SES		-	0.03	0.28 **	0.15	0.02	0.12	0.1	0.11	0.11	0.01	0.21 *	−0.13	−0.24 **
3. GnG–Go–CR			-	−0.31 ***	−0.07	0.22 *	0.42 ***	0.01	0.22 *	0.14	0.25 **	0.40 ***	−0.27 **	0.16
4. GnG–T				-	0.49 ***	0.18 *	0.18 *	0.31 ***	0.02	0.19 *	0.07	0.04	−0.08	−0.04
5. GnG–NoGo–CR					-	0.23 *	0.31 ***	0.23 *	0.15	0.15	0.21 *	0.39 ***	−0.15	0.12
6. DCCS–CR						-	0.27 **	0.23 *	0.23 *	0.38 ***	0.35 ***	0.32 ***	−0.19 *	0.17
7. FC–CR							-	0.29 **	0.31 ***	0.33 ***	0.35 ***	0.37 ***	−0.39 ***	0.18 *
8. FC–T								-	0.1	0.73 ***	0.17	0.1	−0.21 *	0.07
9. FI–CR									-	0.17	0.31 ***	0.18 *	−0.15	0.08
10. FI–T										-	0.24 *	0.17	−0.33 ***	0.12
11. MG–CR											-	0.36 ***	−0.22 *	0.04
12. IPDA score												-	−0.33 ***	0.22 *
13. SR-SA score													-	−0.17
14. EFB score														-

Legend: SES: Socio-economic status; CR = correct responses; T = median time; GnG = Go–NoGo test; Go = Go condition; NoGo = NoGo condition; DCCS = Dimensional Change Card Sort Test Shape condition; FC = Flanker test congruous condition; FI = Flanker test incongruous condition; MG = Mr. Giraffe test; SR-SA = Self-regulation behavior during structured activities; EFB: Executive function behavior; * *p* < 0.05; ** *p* < 0.01; *** *p* < 0.001.

**Table 4:** Regression coefficients for all EF predictors and covariates on the learning prerequisites (IPDA scores).

Measures	B	SEB	β	VIF
Age in months	0.85	0.44	0.2	1.91
SES	4.39	2.07	0.17	1.16
GnG–Go–CR	1.17	0.41	0.29	1.94
GnG–T	−0.02	0.03	−0.08	2.02
GnG–NoGo–CR	2.63	0.77	0.32	1.6
DCCS–CR	3.27	1.9	0.15	1.34
FC–CR	0.25	0.63	0.04	2.11
FC–T	−2.76	0.01	0.00	2.63
FI–CR	−0.59	0.46	−0.11	1.35
FI–T	−0.01	0.01	−0.11	2.71
MG–CR	2.4	1.1	0.19	1.38

Legend: CR = correct responses; T = median time; GnG = Go–NoGo test; Go = Go condition; NoGo = NoGo condition; DCCS = Dimensional Change Card Sort test Shape condition; FC = Flanker test congruous condition; FI = Flanker test incongruous condition; MG = Mr. Giraffe test; SES: Socio-economic Status.

## Data Availability

The data presented in this study are available on request from the corresponding author.
